# Immunogenicity and Safety of Extended Dosing Intervals for Pfizer Pentavalent MenABCWY Meningococcal Vaccination in Healthy Adolescents: Results from a Randomized, Phase 2b Study

**DOI:** 10.3390/vaccines14040352

**Published:** 2026-04-15

**Authors:** Jake C. Jones, Mary D. Tipton, Lefteris Zolotas, Jason D. Maguire, Kelly Belanger, Yanping Liu, Roger Maansson, Robert E. O’Neill, Paul Balmer, Paula Peyrani, Johannes Beeslaar

**Affiliations:** 1 Wasatch Pediatrics, Murray, UT 84107, USA; 2 CopperView Medical Center, South Jordan, UT 84095, USA; 3 Pfizer Vaccine Research and Development, Marlow SL7 1YL, UK; 4 Pfizer Vaccine Research and Development, Collegeville, PA 19426, USA; 5 Pfizer Vaccine Research and Development, Pearl River, NY 10965, USA; 6 Pfizer US Vaccines Medical Affairs, Collegeville, PA 19426, USA

**Keywords:** vaccines, invasive meningococcal disease, MenABCWY, vaccination schedule, adolescents, dosing interval, immunogenicity, safety

## Abstract

**Background/Objectives:** Meningococcal disease is primarily caused by serogroups A, B, C, W, and Y. Current US vaccination recommendations include routine serogroup A/C/W/Y (MenACWY) vaccination (ages 11–12 and 16 years) and a two-dose, 0-, 6-month MenB vaccination series (age 16–23 years) based on shared clinical decision-making. Administration of the first-in-class Pfizer pentavalent MenABCWY vaccine (Penbraya^TM^), which received US licensure in 2023 as a two-dose, 0-, 6-month series, is endorsed when the MenACWY and MenB vaccines are recommended at the same visit. This study evaluated the immunogenicity and safety of two extended two-dose schedules of MenABCWY in healthy adolescents. **Methods:** In this observer-blinded, phase 2b study (ClinicalTrials.gov, NCT04440176; 19 June 2020), 309 healthy 11- to 14-year-olds were randomized 1:1 to receive a 0-, 36-month or 0-, 12-month Pfizer MenABCWY schedule, which more closely aligns with current US MenACWY vaccination recommendations. Endpoints included serum bactericidal assay using human complement seroprotection rates (titers ≥ 1:8 or ≥1:16, depending on strain), seroresponse rates (≥4-fold increase from baseline titer), and geometric mean titers (GMTs). Safety was also assessed. **Results:** One month after the second Pfizer MenABCWY dose, serogroup A/B/C/W/Y seroprotection rates were 100% for the 0-, 36-month schedule and 96.6–100% for the 0-, 12-month schedule; seroresponse rates were 100% and 92.9–100%, respectively. GMTs generally trended higher with the 0-, 36-month schedule. Seroprotection rates through 24 months after the second dose of the 0-, 12-month schedule were 44.0–75.0% for serogroup B and 88.9–100% for serogroup A/C/W/Y). No safety issues were identified. **Conclusions:** These data support Pfizer MenABCWY dosing flexibility and utility within the current or possible future US meningococcal vaccination framework.

## 1. Introduction

Invasive meningococcal disease (IMD) is caused by the bacterium *Neisseria meningitidis* and is associated with high morbidity and mortality [1]. Although IMD initially presents as nonspecific symptoms, it quickly progresses to life-threatening manifestations, including septicemia and meningitis [2,3]. The overall mortality rate is estimated at 8% [4], and many survivors experience serious, long-term sequelae, such as hearing loss, cognitive impairment, or limb amputation [5]. Disease incidence is highest among infants and young children, followed by adolescents and young adults and, in many regions, older adults [6,7].

Five meningococcal serogroups (A, B, C, W, and Y) have historically caused the vast majority of IMD globally [8]. Until recently, available meningococcal vaccines included polysaccharide conjugate vaccines for serogroups A, C, W, and Y (MenACWY) and separate, protein-based vaccines for meningococcal serogroup B (MenB) [8]. In October 2023, a pentavalent MenABCWY vaccine developed by Pfizer (Penbraya^TM^; Pfizer Ireland Pharmaceuticals, Ringaskiddy, Cork, Ireland) and composed of two licensed vaccines—bivalent factor H binding protein (fHbp) MenB vaccine (MenB-fHbp, Trumenba^®^; Pfizer Inc, Philadelphia, PA, USA) and tetanus toxoid-conjugate MenACWY vaccine (MenACWY-TT, Nimenrix^®^; Pfizer Europe MA EEIG, Brussels, Belgium)—became the first of its kind to receive licensure from the US Food and Drug Administration [9]; as a combination vaccine, the Pfizer MenABCWY vaccine may help simplify the current landscape of IMD prevention [10]. The Pfizer MenABCWY vaccine is licensed for adolescents and young adults 10 through 25 years of age as a two-dose series, with doses administered 6 months apart [11].

The US Advisory Committee on Immunization Practices (ACIP) currently recommends routine administration of a single MenACWY conjugate vaccine dose at age 11 to 12 years plus a booster dose at age 16 years and administration of a two-dose MenB vaccine series at age 16 to 23 years (16–18 years preferred) based on shared clinical decision-making [12]. Consistent with the stronger recommendation for MenACWY versus MenB vaccination, approximately 91% of 17-year-olds in the United States received ≥1 dose of the MenACWY vaccine in 2023, whereas about 32% received ≥1 dose of the MenB vaccine [13]. Following licensure of the Pfizer MenABCWY vaccine, the ACIP recommended that adolescents may receive a single Pfizer MenABCWY dose as an alternative to concurrent administration of MenACWY and MenB vaccine [12,14].

Adolescents and young adults face unique barriers to vaccination, including lack of knowledge and inadequate communication between healthcare providers and adolescents, lack of primary healthcare visits, and limited opportunities for vaccination [15]. The ability to take advantage of these limited opportunities can be maximized by vaccines allowing flexible schedules, such that similar tolerability and protective responses are observed across schedules comprising a wide range of intervals between doses. Additionally, the option for an extended interval between vaccination doses would give healthcare providers the flexibility to tailor vaccination to local epidemiology and existing vaccination platforms.

To potentially simplify and provide flexibility for vaccine scheduling within the current landscape of IMD prevention, we sought to investigate the safety and immunogenicity of extended interval schedules for the Pfizer pentavalent MenABCWY vaccine. These evaluations will inform the potential for flexible scheduling of Pfizer MenABCWY vaccination and its use within the current or possible future US meningococcal vaccination framework.

## 2. Materials and Methods

### 2.1. Study Design, Participants, and Procedures

This phase 2b, randomized, observer-blinded, multicenter study (ClinicalTrials.gov, NCT04440176, registered 19 June 2020) took place between June 2020 and January 2024 across 21 sites in the United States. Participants were randomized by study site personnel using an interactive response technology (IRT) system in a 1:1 ratio to receive the Pfizer MenABCWY vaccine using either a 0-, 12-month or 0-, 36-month schedule (i.e., Month 0, 12 and Month 0, 36 groups, respectively; Figure S1). The site personnel entered information, including the participant’s protocol number and date of birth, into the IRT system, which generated a participant randomization number that was recorded on the case report form and used as an identifier prior to unblinding. Personnel who enrolled and those who assigned participants to the interventions were also blinded, whereas the study staff who dispensed, prepared, and administered the vaccine were unblinded. To maintain blinding to study intervention assignment for all other study and site personnel as well as the investigator and investigator’s staff and the participants and their parent(s)/guardian(s), the Month 0, 36 participants received placebo at Month 12.. In accordance with the study protocol, unblinding occurred at 1 month after all participants completed the second vaccination visit (i.e., Month 13 for both groups, which was 1 month after the second Pfizer MenABCWY dose for the Month 0, 12 group and 13 months after the first Pfizer MenABCWY dose for the Month 0, 36 group). Participants in the Month 0, 36 group received the second Pfizer MenABCWY dose at Month 36 (i.e., at the third vaccination visit, after receipt of placebo at the second vaccination visit) in an open-label manner.

Eligible participants were healthy, meningococcal vaccine-naive individuals who were 11 to 14 years of age at the time of randomization and were able and willing to comply with study procedures. Key exclusion criteria included immunodeficiency or immunosuppression, or a history of microbiologically proven disease caused by *N meningitidis* or *N gonorrhoeae*.

The Pfizer MenABCWY vaccine and saline placebo were administered via intramuscular injection (0.5 mL per dose) into the upper deltoid of the right or left arm. To maintain blinding, study vaccine syringes at Months 0 and 12 were administered in a manner that prevented group identification based on the differing physical appearance of the Pfizer MenABCWY vaccine and placebo. Blood draws for immunogenicity assessments were performed before the first dose and 1 month after each vaccination visit; participants in the Month 0, 12 group also had blood drawn for immunopersistence evaluations at 12 and 24 months after the second Pfizer MenABCWY dose.

The study complied with the ethical principles of the Declaration of Helsinki and International Council for Harmonisation of Technical Requirements for Pharmaceuticals for Human Use Good Clinical Practice Guidelines. Local regulatory requirements were adhered to, and the final protocol, any amendments, and informed consent documents were approved by institutional review boards (Advarra [central institutional review board; reference number Pro00043007, approved 3 April 2020] and the Cincinnati Children’s Hospital Institutional Review Board [reference number 2020-0484, approved 2 September 2020]). Written informed assent by all participants, as required by local regulations, and consent by their parents or guardians, was documented.

### 2.2. Study Objectives and Endpoints

The primary immunogenicity objective for each of the Month 0, 12 and Month 0, 36 groups was to assess the serogroup B immune responses after 2 Pfizer MenABCWY doses. Serogroup B immune responses after a single dose were not assessed because a single dose is not expected to provide adequate protection, as reflected in the approved 2-dose, 0-, 6-month posology for the Pfizer MenABCWY and MenB-fHbp vaccines [11,16]. Serogroup B immune responses were evaluated in terms of the percentage of participants with seroprotective serum bactericidal assay using human complement (hSBA) titers against each of 4 primary serogroup B test strains measured at baseline and 1 month after the second Pfizer MenABCWY dose. The primary serogroup B test strains express vaccine-heterologous fHbp variants (A22, A56, B24, and B44); these strains were selected to demonstrate breadth of coverage against diverse serogroup B disease-causing strains and have been used for serogroup B immunogenicity assessments in previous Pfizer MenABCWY and MenB-fHbp licensure studies [17,18]. Seroprotective titers were defined as hSBA titers greater than or equal to the lower limit of quantitation (LLOQ), which was defined as a titer of 1:16 for the test strain expressing fHbp variant A22 and a titer of 1:8 for the remaining 3 test strains (i.e., more stringent than the accepted correlate of protection of 1:4 [19]).

A secondary immunogenicity objective for each group was to assess the immune responses against serogroups A, C, W, and Y after each Pfizer MenABCWY dose, and was evaluated as the percentage of participants with seroprotective hSBA titers against serogroups A, C, W, and Y measured at baseline and 1 month after each Pfizer MenABCWY dose; LLOQs for each of these test strains were defined as hSBA titers of 1:8. An additional secondary immunogenicity objective for the Month 0, 12 group was to describe the persistence of the immune response against each of the 5 serogroups, as measured by the percentage of participants with seroprotective titers against each test strain at 12 and 24 months after the second Pfizer MenABCWY dose.

Exploratory immunogenicity endpoints for both groups included the percentage of participants with an hSBA seroresponse, which was defined as a ≥4-fold rise in titers from baseline for participants with baseline hSBA titers ≥ LLOQ, a titer of ≥1:16 for participants with baseline hSBA titers < 1:4, and a titer ≥ 4 times the LLOQ for participants with baseline hSBA titers ≥ 1:4 and <LLOQ. Seroresponses were evaluated for each of the primary serogroup B and serogroup A, C, W, and Y test strains; for serogroup B, an additional exploratory endpoint was the percentage of participants with composite responses, defined as hSBA titers ≥ LLOQ against all 4 serogroup B test strains combined. As a supplemental analysis, the lower limit of the 95% CIs (LCIs) for each of the seroresponse and composite response rates at 1 month after the second Pfizer MenABCWY dose in the Month 0, 36 group were compared with prespecified thresholds (Table S1); for serogroup B endpoints, these thresholds were overall more stringent than those used to provide support for the 0-, 6-month Trumenba^®^ (MenB-fHbp) licensing criteria agreed on with the Food and Drug Administration Center for Biologics Evaluation and Research [20]. Additional exploratory immunogenicity endpoints for both study groups were geometric mean titers (GMTs) against each of the primary serogroup B and serogroup A, C, W, and Y test strains. Exploratory endpoints for serogroups A, C, W, and Y were evaluated at baseline (if applicable) and 1 month after each Pfizer MenABCWY dose, whereas those for serogroup B were evaluated at baseline (if applicable) and 1 month after the second Pfizer MenABCWY dose.

The primary safety objective was to describe the safety profile of the 0-, 12-month and 0-, 36-month Pfizer MenABCWY dosing schedules. Corresponding endpoints included the percentage of participants reporting adverse events (AEs), serious AEs (SAEs), medically attended AEs (MAEs), and newly diagnosed chronic medical conditions (NDCMCs) from each vaccination through 1 month after each vaccination; rates of SAEs, MAEs, and NDCMCs occurring from the first vaccination through 6 months after the first vaccination (i.e., Month 0; Pfizer MenABCWY for both groups) and from the second vaccination through 6 months after the second vaccination (i.e., Month 12; Pfizer MenABCWY for the Month 0, 12 group and placebo for the Month 0, 36 group), were also evaluated. Prespecified reactogenicity events were not solicited in this study (i.e., no electronic diaries were used).

### 2.3. Statistical Methods and Analysis Populations

All statistical analyses of immunogenicity and safety were descriptive. The study planned to enroll approximately 150 participants in each vaccine group and, assuming an up to 20% exclusion rate from the applicable evaluable population, was expected to yield approximately 120 evaluable participants per group. At 120 participants per group, the expected half-width of 95% CIs (i.e., margin of error) by percentage of participants achieving an hSBA titer ≥ LLOQ for each test strain was calculated to be 9.1% and 5.8% at 60% and 90% of participants, respectively. The probability of observing ≥ 1 occurrence of any AE for true event percentages at 0.1%, 0.5%, 1.0%, and 2.0% when MenABCWY is administered to 150 participants is 0.14, 0.53, 0.78, and 0.95, respectively. Immunogenicity endpoints were assessed in the evaluable immunogenicity populations, which, for each Pfizer MenABCWY dose, included all randomized participants who maintained eligibility without any major protocol deviations through 1 month after the Pfizer MenABCWY dose; received the first Pfizer MenABCWY dose (post–dose 1 evaluable immunogenicity population) or both Pfizer MenABCWY doses (post–dose 2 evaluable immunogenicity population) as randomized; had blood drawn for assay testing within the required times at baseline and 1 month after the Pfizer MenABCWY dose; and had ≥1 valid and determinate serogroup A, B (second dose only), C, W, or Y assay result at 1 month after the Pfizer MenABCWY dose. Safety endpoints were evaluated in the safety populations corresponding to each vaccination, which included all participants who received vaccination 1 (i.e., Pfizer MenABCWY for both groups) and had any available safety data up to 12 months after vaccination 1 (vaccination 1 safety population), received vaccination 2 (i.e., Pfizer MenABCWY for the Month 0, 12 group and placebo for the Month 0, 36 group) and had any available safety data up to 24 months after vaccination 2 (vaccination 2 safety population), or received vaccination 3 (i.e., Pfizer MenABCWY for the Month 0, 36 group) and had any available safety data up to 1 month after vaccination 3 (vaccination 3 safety population). The safety population included those randomized participants who received ≥1 dose of study intervention and had any available safety data after vaccination.

Data analysis was performed at 2 separate time points. Analysis 1 was performed after all participants completed the visit at 1 month after the Month 12 vaccination and included safety and immunogenicity data through this visit. The final analysis was performed after all available data were collected. Missing immunogenicity results were not imputed and missing AE dates were imputed according to Pfizer safety rules.

Binary immunogenicity and safety endpoints were reported as percentages and associated Clopper–Pearson 95% CIs. For GMT calculations, hSBA titers < LLOQ were set to 0.5 × LLOQ; GMTs were calculated by log transforming assay results, evaluating the mean and associated 95% CIs based on a Student *t* distribution, and exponentiating the results.

## 3. Results

### 3.1. Study Population

**Figure 1:**
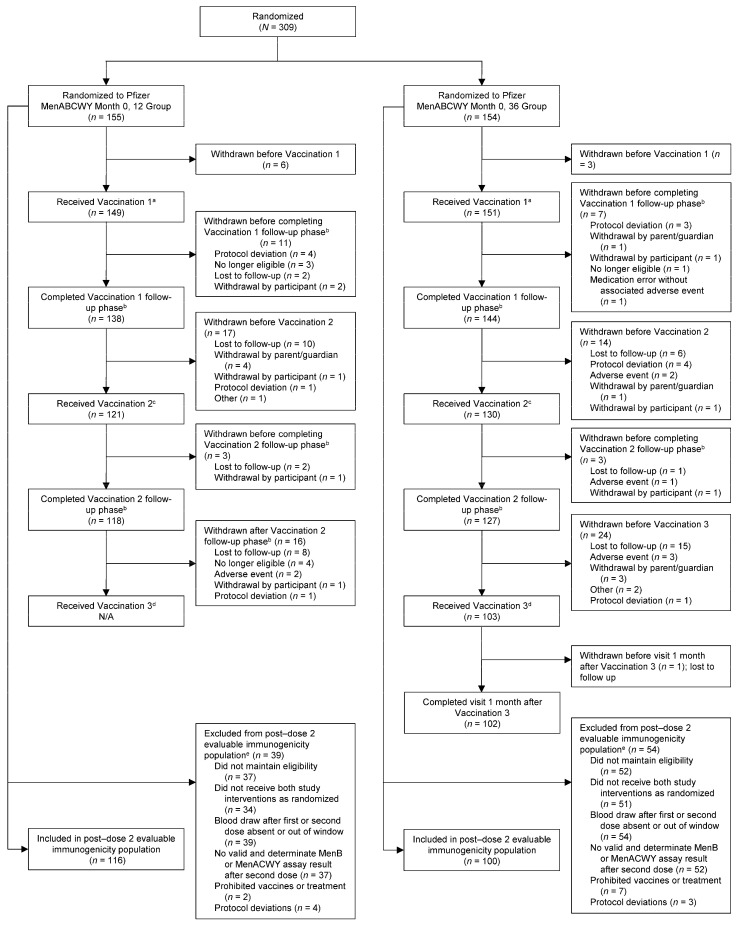
Flow diagram with participant dispositions and analysis populations. ^a^ Vaccination 1 was the first dose of Pfizer MenABCWY for both groups. ^b^ Follow-up phase was defined as from vaccination phase through 6 months (196 days) after vaccination. ^c^ Vaccination 2 was the second dose of Pfizer MenABCWY for the Month 0, 12 group and placebo for the Month 0, 36 group. ^d^ Vaccination 3 was the second dose of Pfizer MenABCWY for the Month 0, 36 group; the Month 0, 12 group did not receive a third vaccination. ^e^ Participants could be excluded from the post-dose 2 evaluable immunogenicity population for more than 1 reason. MenABCWY = meningococcal serogroup A, B, C, W, and Y vaccine; N/A = not applicable.

Participant enrollment spanned from 17 June through 11 December 2020, with follow-up continuing through 5 January 2024. A total of 309 study participants were randomized: 155 to the Month 0, 12 group and 154 to the Month 0, 36 group (Figure 1). In the Month 0, 12 group, 149 (96.1%) and 121 (78.1%) participants received the first and second Pfizer MenABCWY doses, respectively, whereas 151 (98.1%), 130 (84.4%), and 103 (66.9%) participants in the Month 0, 36 group received the first Pfizer MenABCWY dose, placebo vaccination at Month 12, and second Pfizer MenABCWY dose, respectively. The percentage of participants who completed all study visits was similar in the Month 0, 12 group (66.5%) and the Month 0, 36 group (65.6%). The most common reason for study withdrawal across groups was loss to follow-up (*n* = 45 [14.6%]). The post-dose 2 evaluable immunogenicity population included 116 and 100 participants in the Month 0, 12 and Month 0, 36 groups, respectively (Figure 1).

Participant demographics were generally similar across study groups (Table 1). The majority of participants across groups were White (85.4%) and male (55.4%). Median age at first vaccination was 11.0 years. Demographics and baseline characteristics among participants who completed the second Pfizer MenABCWY vaccine were similar to those in the safety population (Table S2). Demographics of participants who completed the study versus did not complete the study were generally similar, although low participant numbers in some subgroups limited conclusions that could be drawn from the comparisons (Table S3).

### 3.2. Immunogenicity

**Figure 2:**
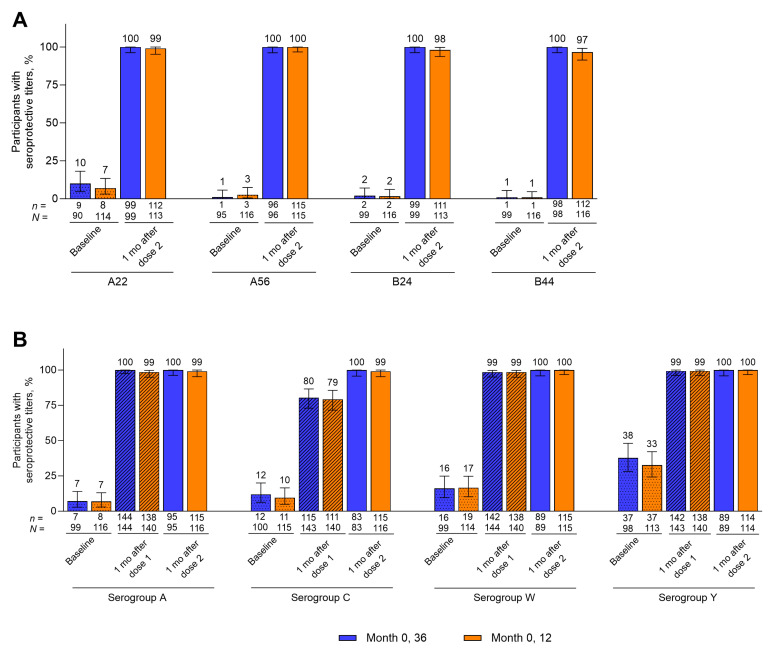
Percentages of participants with seroprotective hSBA titers against (**A**) serogroup B strains and (**B**) serogroup A, C, W, and Y strains through 1 month after the second Pfizer MenABCWY dose. Corresponding data are presented in Table S4, with percentages of participants with hSBA titers ≥1:4 for the serogroup B strains shown for reference. Data for baseline and 1 month after dose 2 are for the post-dose 2 evaluable immunogenicity populations (Month 0, 36 group, *n* = 83–100; Month 0, 12 group, *n* = 113–116). Data for 1 month after dose 1 are for the post-dose 1 evaluable immunogenicity populations (Month 0, 36 group, *n* = 143–144; Month 0, 12 group, *n* = 140). Serogroup B strains are indicated by the vaccine-heterologous fHbp variants they express. Error bars represent 95% CIs. Seroprotective titers were defined as hSBA titers ≥ LLOQ (1:16 for the strain expressing fHbp variant A22; 1:8 for all other strains). Blue bars, Month 0, 36; orange bars, Month 0, 12. fHbp = factor H binding protein; hSBA = serum bactericidal assay using human complement; LLOQ = lower limit of quantitation; MenABCWY = meningococcal serogroup A, B, C, W, and Y vaccine.

At baseline, ≤10.0% of participants in each group had seroprotective hSBA titers against each of the 4 primary serogroup B test strains (Figure 2A, Table S4). Serogroup B hSBA seroprotection rates at 1 month after the second Pfizer MenABCWY dose rose to 100% for all strains in the Month 0, 36 group and to a range of 96.6% to 100% in the Month 0, 12 group (Figure 2A, Table S4). For serogroups A, C, W, and Y, hSBA seroprotection rates at baseline ranged from 6.9% to 37.8% across groups; percentages at 1 month after the second Pfizer MenABCWY dose were 100% in the Month 0, 36 group and 99.1% to 100% in the Month 0, 12 group (Figure 2B, Table S4). hSBA seroprotection rates against serogroups A, C, W, and Y at 1 month after the first Pfizer MenABCWY dose were comparable across the Month 0, 36 and Month 0, 12 groups (Figure 2B, Table S4).

**Figure 3:**
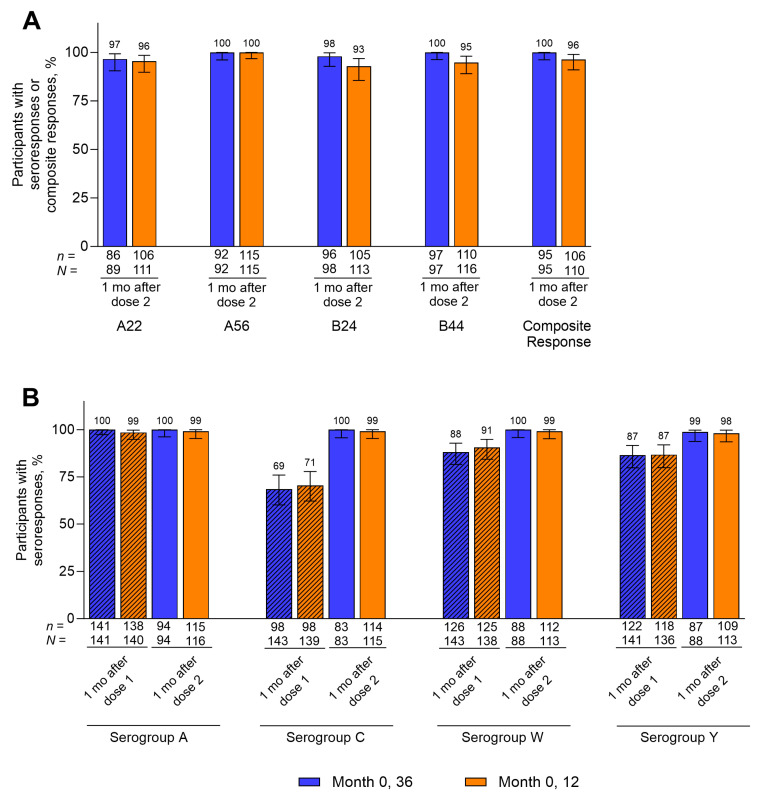
Percentages of participants with hSBA (**A**) seroresponses and composite responses against serogroup B strains and (**B**) seroresponses against serogroup A, C, W, and Y strains through 1 month after the second Pfizer MenABCWY dose. Corresponding data are in Table S5. Data for 1 month after dose 2 are for the post-dose 2 evaluable immunogenicity populations (Month 0, 36 group, *n* = 83–98; Month 0, 12 group, *n* = 110–116). Data for 1 month after dose 1 are for the post-dose 1 evaluable immunogenicity populations (Month 0, 36 group, *n* = 141–143; Month 0, 12 group, *n* = 136–140). Serogroup B strains are indicated by the vaccine-heterologous fHbp variants they express. Error bars represent 95% CIs. For participants with baseline hSBA titers < 1:4, seroresponse was defined as a titer of ≥1:16; for participants with baseline hSBA titers ≥ 1:4 and <LLOQ (1:16 for the strain expressing fHbp variant A22; 1:8 for all other strains), seroresponse was defined as a titer ≥4 times the LLOQ; and for participants with baseline hSBA titers ≥ LLOQ, seroresponse was defined as a ≥4-fold rise in titer from baseline. Composite responses were evaluated for serogroup B only and were defined as seroprotective titers (titers ≥ LLOQ) for all 4 serogroup B strains combined. Blue bars, Month 0, 36; orange bars, Month 0, 12. fHbp = factor H binding protein; hSBA = serum bactericidal assay using human complement; LLOQ = lower limit of quantitation; MenABCWY = meningococcal serogroup A, B, C, W, and Y vaccine.

At 1 month after the second dose, 96.6% to 100% of participants in the Month 0, 36 group and 92.9% to 100% of participants in the Month 0, 12 group achieved hSBA seroresponses across the four primary serogroup B test strains (Figure 3A, Table S5). Composite response rates were 100% and 96.4% in the Month 0, 36 and Month 0, 12 groups, respectively. Percentages of participants with hSBA seroresponses against serogroups A, C, W, and Y at 1 month after the second dose were 98.9% to 100% in the Month 0, 36 group and 98.2% to 99.1% in the Month 0, 12 group (Figure 3B, Table S5). For all seroresponse rates and the composite response rate at 1 month after the second Pfizer MenABCWY dose in the Month 0, 36 group, the LCI exceeded the corresponding prespecified thresholds (Table S1). Seroresponse rates against serogroups A, C, W, and Y at 1 month after the first Pfizer MenABCWY dose were generally comparable across both schedules (Figure 3B, Table S5).

**Figure 4:**
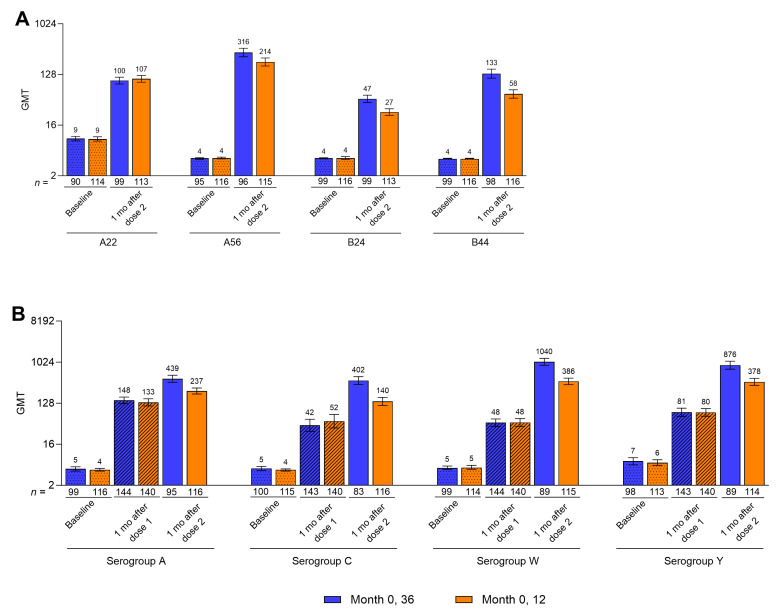
hSBA GMTs among participants against (**A**) serogroup B strains and (**B**) serogroup A, C, W, and Y strains through 1 month after the second Pfizer MenABCWY dose. Corresponding data are in Table S6. Data for baseline and 1 month after dose 2 are for the post-dose 2 evaluable immunogenicity populations (Month 0, 36 group, *n* = 83–100; Month 0, 12 group, *n* = 113–116). Data for 1 month after dose 1 are for the post-dose 1 evaluable immunogenicity populations (Month 0, 36 group, *n* = 143–144; Month 0, 12 group, *n* = 140). Serogroup B strains are indicated by the vaccine-heterologous fHbp variants they express. Error bars represent 95% CIs. Blue bars, Month 0, 36; orange bars, Month 0, 12. fHbp = factor H binding protein; GMT = geometric mean titer; hSBA = serum bactericidal assay using human complement; MenABCWY = meningococcal serogroup A, B, C, W, and Y vaccine.

For all five serogroups, hSBA GMTs increased substantially from baseline to 1 month after the second dose for both the Pfizer MenABCWY 0-, 36-month and 0-, 12-month schedules (Figure 4, Table S6). Comparisons across groups indicated that hSBA GMTs at 1 month after the second Pfizer MenABCWY dose generally trended higher as the dosing interval increased from 12 to 36 months. Specifically, hSBA GMTs against serogroup B strains ranged from 47.0 to 315.6 in the Month 0, 36 group and from 27.3 to 213.7 in the Month 0, 12 group. Corresponding GMTs against serogroups A, C, W, and Y ranged from 401.9 to 1040.1 and from 140.0 to 385.7. hSBA GMTs against serogroups A, C, W, and Y at 1 month after the first Pfizer MenABCWY dose were generally similar for both schedules (Figure 4B, Table S6).

**Figure 5:**
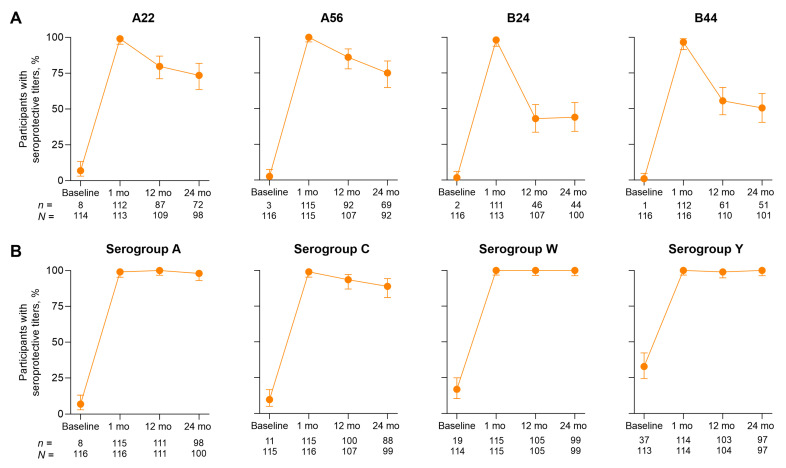
Percentages of participants with seroprotective hSBA titers against (**A**) serogroup B strains and (**B**) serogroup A, C, W, and Y strains through 24 months after the second Pfizer MenABCWY dose for the Month 0, 12 group. Data are for the post-dose 2 evaluable immunogenicity population (*n* = 92–116). Serogroup B strains are indicated by the vaccine-heterologous fHbp variants they express. Error bars represent 95% CIs. Seroprotective titers were defined as hSBA titers ≥LLOQ (1:16 for the strain expressing fHbp variant A22; 1:8 for all other strains). fHbp = factor H binding protein; hSBA = serum bactericidal assay using human complement; LLOQ = lower limit of quantitation; MenABCWY = meningococcal serogroup A, B, C, W, and Y vaccine.

Antibody persistence evaluations showed that 44.0% to 75.0% of participants in the Month 0, 12 group retained seroprotective titers against serogroup B strains at 24 months after the last dose; for serogroups A, C, W, and Y, percentages were 88.9% to 100% (Figure 5).

### 3.3. Safety

Adverse events were reported by 29.1% (*n* = 43) and 30.8% (*n* = 45) of participants in the Month 0, 36 and Month 0, 12 groups, respectively, within 1 month after the first Pfizer MenABCWY dose (Table 2). Reporting rates within 1 month after the second Pfizer MenABCWY dose were 19.4% (*n* = 20) and 15.7% (*n* = 19), respectively. Severe AEs were reported by one participant in the Month 0, 36 group after each Pfizer MenABCWY dose (0.7% and 1.0%, respectively) and by three (2.1%) participants in the Month 0, 12 group after the first dose; all were attributable to reactogenicity-type events. Most of the vaccine-related AEs, per investigators’ assessment, reported within 1 month after the first (Month 0, 36 group, 22.3% [*n* = 33]; Month 0, 12 group, 19.2% [*n* = 28]) and second (14.6% [*n* = 15] and 7.4% [*n* = 9], respectively) Pfizer MenABCWY doses were attributable to reactogenicity-type events, such as injection site pain, injection site erythema, pyrexia, headache, fatigue, vomiting, and chills.

Reported MAEs (Month 0, 36 group, 19.6% [*n* = 29] within 6 months after the first Pfizer MenABCWY dose and 3.9% [n = 4] within 1 month after the second Pfizer MenABCWY dose; Month 0, 12 group, 23.3% [*n* = 34] and 13.2% [*n* = 16] within 6 months of the first and second Pfizer MenABCWY doses, respectively; Table 2) were generally consistent with events expected in the study age group, such as injuries and infections. A single participant in the Month 0, 12 group experienced MAEs of sinus tachycardia, vomiting, body aches, and headaches occurring on the day of the first Pfizer MenABCWY dose, which were considered by the study investigator to be possibly related to vaccination. The vomiting and sinus tachycardia in this participant resolved the same day, with the sinus tachycardia resolving within one hour and fifteen minutes of onset; the body aches and headaches resolved within two days. Additionally, an MAE of restless leg syndrome that commenced 140 days after the first Pfizer MenABCWY dose in a participant in the Month 0, 36 group was considered by the study investigator to be possibly related to the Pfizer MenABCWY vaccine or to other nonstudy vaccines (tetanus–diphtheria–pertussis, human papilloma virus, varicella-zoster virus, and influenza) that the participant received between 40 and 112 days before AE onset. Neurologic, musculoskeletal, and laboratory assessments of the participant were normal, and the event resolved within approximately 3 months.

Two participants in the Month 0, 36 group reported a total of three SAEs (one case each of cardiac palpitations, chest pain, and suicidal ideation), all of which occurred within 6 months after the placebo vaccination at Month 12 (Table 2). In the Month 0, 12 group, reported SAEs comprised one case of orbital cellulitis within 6 months after the first Pfizer MenABCWY dose and one case each of acute hepatic failure and intentional overdose, both in the same participant, within 6 months after the second Pfizer MenABCWY dose (Table 2). Details of SAEs of psychiatric conditions are presented in Table S7. A total of nine participants reported 13 NDCMCs during the study reporting period. NDCMCs reported within 6 months of the first Pfizer MenABCWY dose included abdominal pain syndrome and chronic sinusitis in a single participant in the Month 0, 36 group and one report each of scoliosis and depression in the Month 0, 12 group. Six additional participants reported nine NDCMCs of attention deficit hyperactivity disorder (two events), depression (two events), generalized anxiety disorder (two events), major depression, celiac disease, and central auditory processing disorder within 6 months of receiving placebo (administered 12 months after the first Pfizer MenABCWY dose). Overall, the majority of NDCMCs were within the system organ class of psychiatric disorders and most participants reporting NDCMCs of mental health or behavioral issues had associated symptoms starting before study enrollment, a pertinent family history, or a specific trigger associated with the event. None of the reported SAEs or NDCMCs were considered by the investigator to be related to vaccination.

## 4. Discussion

Administration of the Pfizer MenABCWY vaccine according to 0-,36-month and 0-, 12-month schedules in this study induced robust bactericidal immune responses against all five serogroups. For seroprotection and seroresponse outcomes, rates were generally similar in the Month 0, 12 and Month 0, 36 groups. Comparison of GMTs for the two schedules indicated that immune responses after the second Pfizer MenABCWY dose generally trended higher as the dosing interval increased from 12 to 36 months for all five serogroups. Available persistence data indicated that seroprotection rates in the Month 0, 12 group remained substantial for serogroup B and high for serogroups A, C, W, and Y through 24 months after the second Pfizer MenABCWY dose. Both extended interval Pfizer MenABCWY schedules were generally safe and well tolerated, with no reports of SAEs or NDCMCs that were considered related to the vaccine.

The previous pivotal phase 3 study of Pfizer MenABCWY demonstrated that administration of the vaccine on a two-dose, 0-, 6-month schedule was safe, tolerable, and induced robust immune responses in individuals 10 to 25 years of age [21]. Both seroprotection and seroresponses across all serogroups at 1 month after the second dose observed in the extended interval schedules reported in this study were comparable to, or trended higher than, those observed with the 0-, 6-month schedule in the pivotal phase 3 study (Table S8). Persistence of seroprotection through 24 months after the second dose of the 0-, 12-month schedule also appear comparable to, or higher than, those observed with the 0-, 6-month schedule (Table S9). GMTs across all serogroups at 1 month after the second dose for both extended interval schedules reported in this study also appear to trend higher compared with those GMTs observed for the 0-, 6-month schedule (Table S8).

Adolescents are a particularly difficult target population for immunizations and often have low compliance rates because of infrequent routine healthcare visits and missed opportunities for vaccination [15]. These challenges necessitate vaccination schedules that are flexible and can be adjusted based on when vaccination opportunities may arise. Thus, it is encouraging that data collected in this study from adolescents vaccinated on a 0-, 36- or 0-, 12-month schedule, considered alongside results from previous studies evaluating a 0-, 6-month schedule [18,21], suggest that a two-dose Pfizer MenABCWY series can offer protection against all five serogroups across flexible schedules with regard to intervals between vaccinations.

Another means of optimizing prevention against meningococcal disease among adolescents despite limited opportunities for vaccination is reducing the number of immunizations required without compromising breadth of protection [22]. Within the context of the existing US meningococcal vaccination schedule [12], results from the pivotal phase 3 study previously demonstrated immunologic noninferiority of the Pfizer MenABCWY vaccine to separate administration of MenB and MenACWY vaccines [21], suggesting that a single Pfizer MenABCWY vaccine dose can potentially replace separate MenACWY and MenB vaccinations recommended in later adolescence (Figure S2). As mentioned, the ACIP currently endorses use of the Pfizer MenABCWY vaccine as an alternative to concurrent administration of separate MenACWY and MenB vaccines at the same visit [14]. Results from the present study support extending the Pfizer MenABCWY dosing interval such that the first dose is administered in earlier adolescence to provide protection against serogroups A, C, W, and Y in place of the first MenACWY dose recommended at age 11 to 12 years [12], and to prime the immune response against serogroup B to enable boosting in later adolescence during the period of increased risk [23]; the second Pfizer MenABCWY dose will also boost responses against serogroups A, C, W, and Y (Figure S2). In addition to general alignment with the current US vaccination framework and age-specific serogroup incidence, the multiple benefits offered by this approach include fewer injections, which may lead to increased uptake, as well as potentially higher immune responses compared with the currently recommended 6-month MenB vaccine dosing interval.

The trend toward greater serogroup B immune responses with increasing dosing intervals in this study is consistent with previously observed trends for MenB-fHbp, in which immune responses to a two-dose schedule increased when the dosing interval was extended from 2 to 6 months [24]. However, dosing intervals greater than 6 months have not previously been evaluated for MenB-fHbp-containing vaccines. Thus, in addition to providing important clinical insight specific to the Pfizer MenABCWY vaccine, data from the current study extend previous observations for MenB-fHbp-containing vaccines regarding the positive correlation between the magnitude of serogroup B immune responses and dosing intervals to intervals as long as 36 months. The immunologic mechanisms underlying this trend have not yet been investigated.

Extended dosing intervals of more than 6 months have also been evaluated for other vaccines. For another pentavalent MenABCWY vaccine that differs in formulation from the Pfizer MenABCWY vaccine evaluated in the current study, immune responses overall increased as dosing intervals for a two-dose schedule were extended from 1 to 6 months; however, immune responses to 0-, 6-month and 0-, 11-month schedules appeared comparable [25]. Additionally, two studies evaluating separate human papillomavirus vaccines found that immune responses to a two-dose vaccination schedule trended higher for 12-month compared with 6-month dosing intervals [26,27].

Strengths of this study include the prospective, randomized, and blinded study design. The primary limitations were that this analysis was descriptive, and the analysis population was relatively small. In the current study, 96 (31%) vaccinated participants (47 [30%] of those randomized to the Month 0, 12 schedule and 49 [32%] of those randomized to the Month 0, 36 schedule) withdrew from the study. Many withdrawals were due to loss to follow-up (45/96 participants [47%]; of which, 22 and 23 had been randomized to the Month 0, 12 and the Month 0, 36 schedules, respectively), reflecting the challenges of participant retention in adolescent studies of long duration. Notably, few participants (8/96; 8%) withdrew due to AEs. Importantly, as indicated above, the demographics of the participants who completed the study and did not complete the study were similar, and the number of participants who completed the study (*n* = 204) was sufficient to provide reliable immunogenicity and safety results. Of note, subgroup analyses indicated that immune responses across all five serogroups were generally similar in the 11- to 14-year age group compared with the overall 10- to 25-year-old population in the pivotal phase 3 study. More broadly, comparisons between different studies should always be interpreted with caution because of numerous other factors, including potential variation in biological assays and exogenous complement lots used with hSBA [28,29]. In the current study, reactogenicity events were unsolicited; therefore, underreporting of such events cannot be excluded. Also, we note that, in the Month 0, 36 group, the second dose of MenABCWY was open-label, and the collection of safety data following the second Pfizer MenABCWY dose was limited to 1 month. However, for the 0-, 6-month MenABCWY vaccination schedule, previous safety evaluation (including solicited reactogenicity events) demonstrated that the vaccine was well tolerated with no safety concerns through 6 months after the second dose [21].

## 5. Conclusions

Administration of the Pfizer pentavalent MenABCWY vaccine on a 0-, 36-month or 0-, 12-month schedule was safe and well tolerated and induced robust immune responses for all five serogroups included in the vaccine. Findings also indicate that extending the Pfizer MenABCWY dosing interval induced immune responses that were similar or trended higher compared with those associated with the 0-, 6-month schedule evaluated in previous studies. Within the framework of current or future US meningococcal vaccination practices, a two-dose Pfizer MenABCWY schedule spanning early and later adolescence would align with age-specific serogroup epidemiology, potentially increase vaccine uptake by requiring fewer doses compared with current recommendations, and possibly lead to greater immune responses compared with the currently recommended 0-, 6-month MenB vaccine dosing schedule.

## Figures and Tables

**Table 1:** Demographics and baseline characteristics (safety population).

	Month 0, 12 Group (*n* ^a^ = 146)	Month 0, 36 Group (*n* ^a^ = 148)	Total (*N* ^a^ = 294)
Sex, *n* (%)			
Male	81 (55.5)	82 (55.4)	163 (55.4)
Female	65 (44.5)	66 (44.6)	131 (44.6)
Race, *n* (%)			
Black or African American	10 (6.8)	14 (9.5)	24 (8.2)
American Indian or Alaska Native	0	1 (0.7)	1 (0.3)
Asian	3 (2.1)	1 (0.7)	4 (1.4)
Native Hawaiian or other Pacific Islander	1 (0.7)	1 (0.7)	2 (0.7)
White	125 (85.6)	126 (85.1)	251 (85.4)
Multiracial	5 (3.4)	5 (3.4)	10 (3.4)
Not reported	2 (1.4)	0	2 (0.7)
Ethnicity, *n* (%)			
Hispanic/Latino	22 (15.1)	32 (21.6)	54 (18.4)
Non-Hispanic/non-Latino	123 (84.2)	116 (78.4)	239 (81.3)
Not reported	1 (0.7)	0	1 (0.3)
Age at vaccination 1, y			
Mean (SD)	11.5 (0.65)	11.5 (0.65)	11.5 (0.65)
Median (range)	11 (11–14)	11 (11–14)	11 (11–14)

^a^ Number of participants included in the overall safety population.

**Table 2:** Summary of AEs.

Analysis Interval (Population) ^a^	Participants Reporting ≥ 1 AE, *n* (%)	
	Month 0, 12 Group	Month 0, 36 Group
Within 1 mo after vaccination 1 (vaccination 1 safety population)	*n* = 146	*n* = 148
Any AE ^b^	45 (30.8)	43 (29.1)
Related AE	28 (19.2)	33 (22.3)
Severe AE	3 (2.1)	1 (0.7)
Any SAE	0 (0.0)	0 (0.0)
Related SAE	0 (0.0)	0 (0.0)
Any MAE	12 (8.2)	13 (8.8)
Related MAE	1 (0.7)	0 (0.0)
Any NDCMC	1 (0.7)	0 (0.0)
Related NDCMC	0 (0.0)	0 (0.0)
Within 6 mo after vaccination 1 (vaccination 1 safety population)	*n* = 146	*n* = 148
Any SAE	1 (0.7)	0 (0.0)
Related SAE	0 (0.0)	0 (0.0)
Any MAE	34 (23.3)	29 (19.6)
Related MAE	1 (0.7)	1 (0.7)
Any NDCMC	2 (1.4)	1 (0.7)
Related NDCMC	0 (0.0)	0 (0.0)
Within 1 mo after vaccination 2 ^c^ (vaccination 2 safety population)	*n* = 121	*n* = 130
Any AE ^b^	19 (15.7)	21 (16.2)
Related AE	9 (7.4)	3 (2.3)
Severe AE	0 (0.0)	0 (0.0)
Any SAE	0 (0.0)	0 (0.0)
Related SAE	0 (0.0)	0 (0.0)
Any MAE	6 (5.0)	9 (6.9)
Related MAE	0 (0.0)	0 (0.0)
Any NDCMC	0 (0.0)	2 (1.5)
Related NDCMC	0 (0.0)	0 (0.0)
Within 6 mo after vaccination 2 ^c^ (vaccination 2 safety population)	*n* = 121	*n* = 130
Any SAE	1 (0.8)	2 (1.5)
Related SAE	0 (0.0)	0 (0.0)
Any MAE	16 (13.2)	19 (14.6)
Related MAE	0 (0.0)	0 (0.0)
Any NDCMC	0 (0.0)	6 (4.6)
Related NDCMC	0 (0.0)	0 (0.0)
Within 1 mo after vaccination 3 ^d^ (vaccination 3 safety population)	N/A	*n* = 103
Any AE ^b^	N/A	20 (19.4)
Related AE	N/A	15 (14.6)
Severe AE	N/A	1 (1.0)
Any SAE	N/A	0 (0.0)
Related SAE	N/A	0 (0.0)
Any MAE	N/A	4 (3.9)
Related MAE	N/A	0 (0.0)
Any NDCMC	N/A	0 (0.0)
Related NDCMC	N/A	0 (0.0)

AE = adverse event; MAE = medically attended adverse event; MenABCWY = meningococcal serogroup A, B, C, W, and Y vaccine; N/A = not applicable; NDCMC = newly diagnosed chronic medical condition; SAE = serious adverse event. ^a^ Safety populations are defined in the text. ^b^ Reactogenicity events were reported as AEs if the investigator determined that the event met the protocol definition of an AE. ^c^ Vaccination 2 was Pfizer MenABCWY for the Month 0, 12 group and placebo for the Month 0, 36 group. ^d^ Vaccination 3 was the second Pfizer MenABCWY dose for the Month 0, 36 group; the Month 0, 12 group did not receive a third vaccination.

## Data Availability

For legal and privacy reasons, upon request, and subject to review, Pfizer will provide the data that support the findings of this study. Subject to certain criteria, conditions and exceptions, Pfizer may also provide access to the related individual de-identified participant data. See https://www.pfizer.com/science/clinical-trials/trial-data-and-results (accessed on 26 November 2025) for more information.

## Supplementary Materials

The following supporting information can be downloaded at https://www.mdpi.com/article/10.3390/vaccines14040352/s1, Figure S1. Study Design Overview. Figure S2. ACIP Meningococcal Vaccination Schedule (July 2025) and Evaluated Schedules Based Around Pfizer MenABCWY With An Extended Dosing Interval. Table S1. Prespecified Lower 95% CIs for Seroresponse ^a^ and Composite Response ^b^ Endpoints. Table S2. Demographics and Baseline Characteristics Among Participants Who Received the Second Dose of the Pfizer MenABCWY Vaccine. Table S3. Demographics and Baseline Characteristics Among Participants Who Completed vs. Did Not Complete the Study. Table S4. Percentages of Participants with Seroprotective ^a^ hSBA Titers and hSBA titers ≥1:4 Against Serogroup B and Serogroups A, C, W, and Y. Table S5. Percentages of Participants with hSBA Seroresponses ^a^ and Composite Responses ^b^ Against Serogroup B and Serogroups A, C, W, and Y. Table S6. hSBA GMTs Against Serogroup B Strains and Serogroups A, C, W, and Y. Table S7. Details of SAEs of Psychiatric Conditions Occurring Within 6 Months of Study Vaccination. Table S8. hSBA GMTs and Percentages of Participants with Seroprotective hSBA Titers, hSBA titers ≥1:4, and hSBA Seroresponses Against Serogroup B and Serogroups A, C, W, and Y for the 0-, 6-Month Pfizer MenABCWY Vaccine Schedule ^a^. Table S9. Percentages of Participants with Seroprotective hSBA Titers^a^ Against Serogroup B and Serogroup A, C, W, and Y Strains Through 24 Months After the Second Vaccine Dose for the 0-, 6-Month Pfizer MenABCWY Vaccine Schedule^b^.

## Abbreviations

The following abbreviations are used in this manuscript:

ACIP	Advisory Committee on Immunization Practices
AE	adverse event
fHbp	factor H binding protein
GMT	geometric mean titer
hSBA	serum bactericidal assay using human complement
IMD	invasive meningococcal disease
IRT	interactive response technology
LCI	lower limit of the 95% confidence interval
LLOQ	lower limit of quantitation
MAE	medically attended adverse event
MenACWY	meningococcal serogroups A, C, W, and Y
MenACWY-TT	tetanus toxoid-conjugate MenACWY vaccine
MenB	meningococcal serogroup B
MenB-fHbp	factor H binding protein MenB vaccine
NDCMC	newly diagnosed chronic medical conditions
SAE	serious adverse event
