# Supplementary material for: Immunogenicity and Safety of Extended Dosing Intervals for Pfizer Pentavalent MenABCWY Meningococcal Vaccination in Healthy Adolescents: Results from a Randomized, Phase 2b Study
Source: Vaccines (Basel). 2026 Apr 15;14(4):352. doi: 10.3390/vaccines14040352 (PMC13120601; doi:10.3390/vaccines14040352)
Supplement: Supplementary file 1 [file vaccines-14-00352-s001.zip › vaccines-4041683_Figure S2.pdf]

Figure S2. ACIP Meningococcal Vaccination Schedules (July 2025) and Evaluated Schedules Based Around Pfizer MenABCWY With An Extended Dosing Interval. ACIP=Advisory Committee on Immunization Practices.

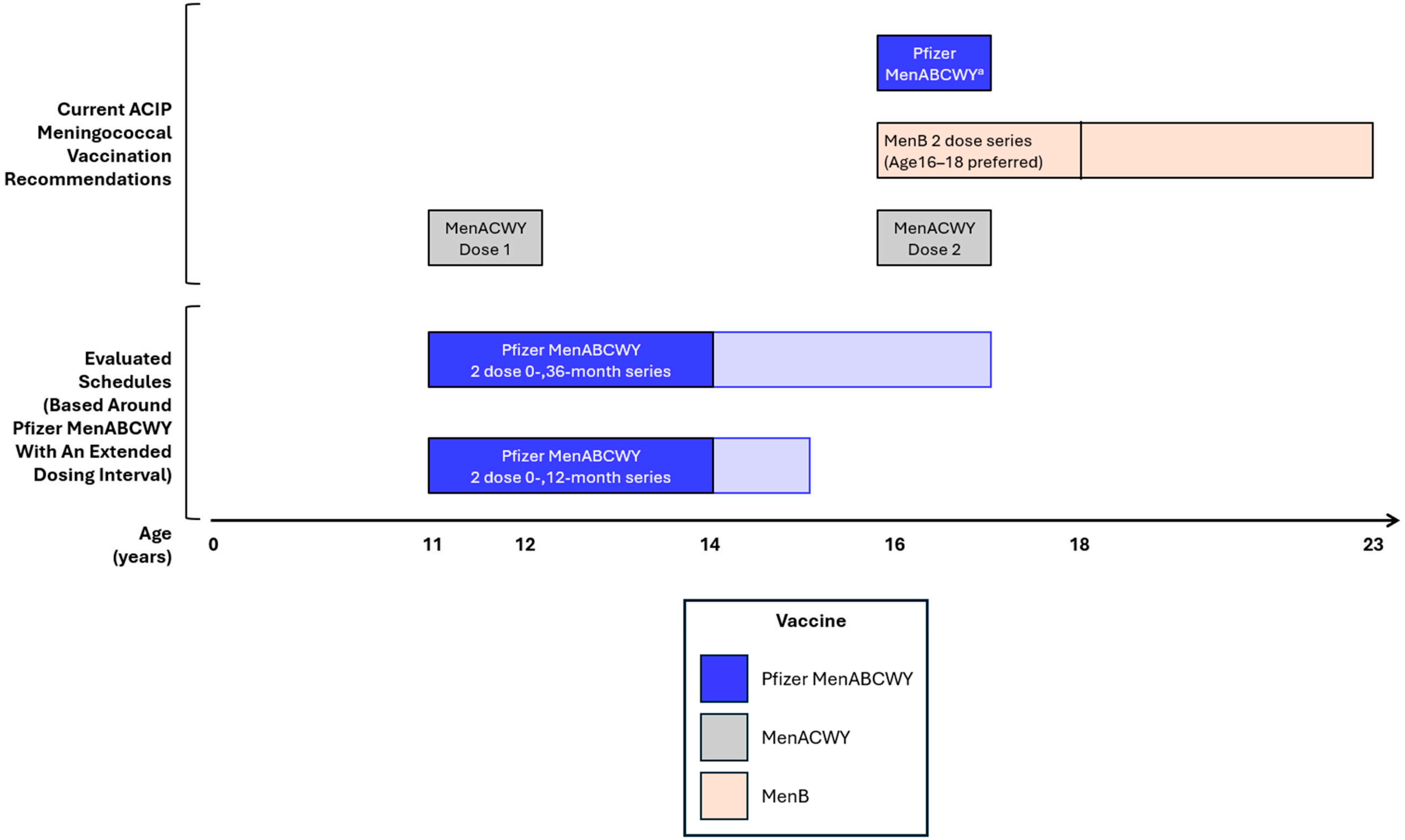

[a]Option when MenACWY and MenB are recommended at same visit.
