# Supplementary material for: Immunogenicity and Safety of Extended Dosing Intervals for Pfizer Pentavalent MenABCWY Meningococcal Vaccination in Healthy Adolescents: Results from a Randomized, Phase 2b Study
Source: Vaccines (Basel). 2026 Apr 15;14(4):352. doi: 10.3390/vaccines14040352 (PMC13120601; doi:10.3390/vaccines14040352)
Supplement: Supplementary file 1 [file vaccines-14-00352-s001.zip › vaccines-4041683_Table S1.pdf]

Table S1. Prespecified Lower 95% CIs for Seroresponse[a] and Composite Response[b] Endpoints

	**Strain[c]**	**Lower 95% CI Threshold**
Serogroup B		
Seroresponse	A22	75%
	A56	85%
	B24	65%
	B44	75%
Composite response	A22, A56, B24, B44 combined	70%
Serogroups A, C, W, Y		
Seroresponse	A	75%
	C	75%
	W	75%
	Y	75%

fHbp=factor H binding protein; hSBA=serum bactericidal assay using human complement; LLOQ=lower limit of quantitation.

[a]For participants with baseline hSBA titers <1:4, seroresponse was defined as a titer of ≥1:16; for participants with baseline hSBA titers ≥1:4 and <LLOQ (1:16 for the strain expressing fHbp variant A22; 1:8 for all other strains), seroresponse was defined as a titer ≥4 times the LLOQ; and for participants with baseline hSBA titers ≥LLOQ, seroresponse was defined as a ≥4-fold rise in titer from baseline.

[b]Composite responses were evaluated for serogroup B only and were defined as seroprotective titers (titers ≥LLOQ) for all 4 serogroup B strains combined.

[c]Serogroup B strains are indicated by the vaccine-heterologous fHbp variants they express.
