# Supplementary material for: Immunogenicity and Safety of Extended Dosing Intervals for Pfizer Pentavalent MenABCWY Meningococcal Vaccination in Healthy Adolescents: Results from a Randomized, Phase 2b Study
Source: Vaccines (Basel). 2026 Apr 15;14(4):352. doi: 10.3390/vaccines14040352 (PMC13120601; doi:10.3390/vaccines14040352)
Supplement: Supplementary file 1 [file vaccines-14-00352-s001.zip › vaccines-4041683_Table S2.pdf]

Table S2. Demographics and Baseline Characteristics Among Participants Who Received the Second Dose of the Pfizer MenABCWY Vaccine

	**Month 0,12 Group (n=121)**	**Month 0,36 Group (n=103)**
Sex, n (%)		
Male	68 (56.2)	61 (59.2)
Female	53 (43.8)	42 (40.8)
Race, n (%)		
Black or African American	8 (6.6)	6 (5.8)
American Indian or Alaska Native	0	1 (1.0)
Asian	3 (2.5)	0
Native Hawaiian or other Pacific Islander	0	1 (1.0)
White	105 (86.8)	91 (88.3)
Multiracial	3 (2.5)	4 (3.9)
Not reported	2 (1.7)	0
Ethnicity, n (%)		
Hispanic/Latino	16 (13.2)	19 (18.4)
Non-Hispanic/non-Latino	104 (86.0)	84 (81.6)
Not reported	1 (0.8)	0
Age at vaccination 1, y		
Mean (SD)	11.5 (0.65)	11.5 (0.65)
Median (range)	11.0 (11.0–14.0)	11.0 (11.0–14.0)
