# Supplementary material for: Immunogenicity and Safety of Extended Dosing Intervals for Pfizer Pentavalent MenABCWY Meningococcal Vaccination in Healthy Adolescents: Results from a Randomized, Phase 2b Study
Source: Vaccines (Basel). 2026 Apr 15;14(4):352. doi: 10.3390/vaccines14040352 (PMC13120601; doi:10.3390/vaccines14040352)
Supplement: Supplementary file 1 [file vaccines-14-00352-s001.zip › vaccines-4041683_Table S3.pdf]

Table S3. Demographics and Baseline Characteristics Among Participants Who Completed vs Did Not Complete the Study

	**Month 0,12 Group**		**Month 0,36 Group**	
	**Completer (n=102)**	**Non-Completer (n=44)**	**Completer (n=102)**	**Non-Completer (n=46)**
Sex, n (%)				
Male	59 (57.8)	22 (50.0)	60 (58.8)	22 (47.8)
Female	43 (42.2)	22 (50.0)	42 (41.2)	24 (52.2)
Race, n (%)				
Black or African American	5 (4.9)	5 (11.4)	6 (5.9)	8 (17.4)
American Indian or Alaska Native	0	0	1 (1.0)	0
Asian	2 (2.0)	1 (2.3)	0	1 (2.2)
Native Hawaiian or other Pacific Islander	0	1 (2.3)	1 (1.0)	0
White	91 (89.2)	34 (77.3)	90 (88.2)	36 (78.3)
Multiracial	3 (2.9)	2 (4.5)	4 (3.9)	1 (2.2)
Not reported	0	1 (2.3)	0	0
Ethnicity, n (%)				
Hispanic/Latino	13 (12.7)	9 (20.5)	18 (17.6)	14 (30.4)
Non-Hispanic/non-Latino	89 (87.3)	34 (77.3)	84 (82.4)	32 (69.6)
Not reported	0	1 (2.3)	0	0
Age at vaccination 1, y				
Mean (SD)	11.5 (0.61)	11.5 (0.73)	11.5 (0.66)	11.5 (0.66)
Median (range)	11.5 (11–14)	11.0 (11–14)	11.0 (11–14)	11.0 (11–14)
