# Supplementary material for: Immunogenicity and Safety of Extended Dosing Intervals for Pfizer Pentavalent MenABCWY Meningococcal Vaccination in Healthy Adolescents: Results from a Randomized, Phase 2b Study
Source: Vaccines (Basel). 2026 Apr 15;14(4):352. doi: 10.3390/vaccines14040352 (PMC13120601; doi:10.3390/vaccines14040352)
Supplement: Supplementary file 1 [file vaccines-14-00352-s001.zip › vaccines-4041683_Table S4.pdf]

*Table S4. Percentages of Participants with Seroprotective[a] hSBA Titers and hSBA titers ≥1:4 Against Serogroup B and Serogroups A, C, W, and Y*

| | Strain[b] | Month 0,36 Group,[a] % (95% CI) | Month 0,36 Group,[c] % (95% CI) | Month 0,12 Group,[a] % (95% CI) | Month 0,12 Group,[c] % (95% CI) |
|---|---|---|---|---|---|
| Serogroup B | | | | | |
| Baseline[d] | A22 | 10.0 (4.7, 18.1) | 12.2 (6.3, 20.8) | 7.0 (3.1, 13.4) | 7.0 (3.1, 13.4) |
| | A56 | 1.1 (0.0, 5.7) | 1.1 (0.0, 5.7) | 2.6 (0.5, 7.4) | 2.6 (0.5, 7.4) |
| | B24 | 2.0 (0.2, 7.1) | 2.0 (0.2, 7.1) | 1.7 (0.2, 6.1) | 1.7 (0.2, 6.1) |
| | B44 | 1.0 (0.0, 5.5) | 1.0 (0.0, 5.5) | 0.9 (0.0, 4.7) | 1.7 (0.2, 6.1) |
| 1 mo after second dose[e] | A22 | 100 (96.3, 100) | 100 (96.3, 100) | 99.1 (95.2, 100) | 99.1 (95.2, 100) |
| | A56 | 100 (96.2, 100) | 100 (96.2, 100) | 100 (96.8, 100) | 100 (96.8, 100) |
| | B24 | 100 (96.3, 100) | 100 (96.3, 100) | 98.2 (93.8, 99.8) | 98.2 (93.8, 99.8) |
| | B44 | 100 (96.3, 100) | 100 (96.3, 100) | 96.6 (91.4, 99.1) | 98.3 (93.9, 99.8) |
| Serogroups A, C, W, Y | | | | | |
| Baseline[f] | A | 7.1 (2.9, 14.0) | - | 6.9 (3.0, 13.1) | - |
| | C | 12.0 (6.4, 20.0) | - | 9.6 (4.9, 16.5) | - |
| | W | 16.2 (9.5, 24.9) | | 16.7 (10.3, 24.8) | - |
| | Y | 37.8 (28.2, 48.1) | - | 32.7 (24.2, 42.2) | - |
| 1 mo after first dose[g] | A | 100 (97.5, 100) | - | 98.6 (94.9, 99.8) | - |
| | C | 80.4 (73.0, 86.6) | - | 79.3 (71.6, 85.7) | - |
| | W | 98.6 (95.1, 99.8) | - | 98.6 (94.9, 99.8) | - |
| | Y | 99.3 (96.2, 100) | - | 99.3 (96.1, 100) | - |
| 1 mo after second dose[h] | A | 100 (96.2, 100) | - | 99.1 (95.3, 100) | - |
| | C | 100 (95.7, 100) | - | 99.1 (95.3, 100) | - |
| | W | 100 (95.9, 100) | - | 100 (96.8, 100) | - |
| | Y | 100 (95.9, 100) | - | 100 (96.8, 100) | |

fHbp=factor H binding protein; hSBA=serum bactericidal assay using human complement; LLOQ=lower limit of quantitation.

Corresponding data are in **Figure 2**.

[a]Seroprotective titers were defined as hSBA titers ≥LLOQ (1:16 for the strain expressing fHbp variant A22; 1:8 for all other strains).

[b]Serogroup B strains are indicated by the vaccine-heterologous fHbp variants they express.

[c]Achieved hSBA titers ≥1:4.

[d]Data are for the post–dose 2 evaluable immunogenicity populations (Month 0,36 group, n=90–99; Month 0,12 group, n=114–116).

[e]Data are for the post–dose 2 evaluable immunogenicity populations (Month 0,36 group, n=96–99; Month 0,12 group, n=113–116).
[f]Data are for the post–dose 2 evaluable immunogenicity populations (Month 0,36 group, n=98–100; Month 0,12 group, n=113–116).
[g]Data are for the post–dose 1 evaluable immunogenicity populations (Month 0,36 group, n=143–144; Month 0,12 group, n=140).
[h]Data are for the post–dose 2 evaluable immunogenicity populations (Month 0,36 group, n=83–95; Month 0,12 group, n=114–116).
