# Supplementary material for: Immunogenicity and Safety of Extended Dosing Intervals for Pfizer Pentavalent MenABCWY Meningococcal Vaccination in Healthy Adolescents: Results from a Randomized, Phase 2b Study
Source: Vaccines (Basel). 2026 Apr 15;14(4):352. doi: 10.3390/vaccines14040352 (PMC13120601; doi:10.3390/vaccines14040352)
Supplement: Supplementary file 1 [file vaccines-14-00352-s001.zip › vaccines-4041683_Table S5.pdf]

Table S5. Percentages of Participants with hSBA Seroresponses[a] and Composite Responses[b] Against Serogroup B and Serogroups A, C, W, and Y

	Strain[c]	Month 0,36 Group, % (95% CI)	Month 0,12 Group, % (95% CI)
Serogroup B			
1 mo after second dose[d]	A22	96.6 (90.5, 99.3)	95.5 (89.8, 98.5)
	A56	100 (96.1, 100)	100 (96.8, 100)
	B24	98.0 (92.8, 99.8)	92.9 (86.5, 96.9)
	B44	100 (96.3, 100)	94.8 (89.1, 98.1)
	Composite	100 (96.2, 100)	96.4 (91.0, 99.0)
Serogroups A, C, W, Y			
1 mo after first dose[e]	A	100 (97.4, 100)	98.6 (94.9, 99.8)
	C	68.5 (60.2, 76.0)	70.5 (62.2, 77.9)
	W	88.1 (81.6, 92.9)	90.6 (84.4, 94.9)
	Y	86.5 (79.8, 91.7)	86.8 (79.9, 92.0)
1 mo after second dose[f]	A	100 (96.2, 100)	99.1 (95.3, 100)
	C	100 (95.7, 100)	99.1 (95.3, 100)
	W	100 (95.9, 100)	99.1 (95.2, 100)
	Y	98.9 (93.8, 99.8)	98.2 (93.6, 99.8)

fHbp=factor H binding protein; hSBA=serum bactericidal assay using human complement; LLOQ=lower limit of quantitation.

Corresponding data are in **Figure 3**.

[a]For participants with baseline hSBA titers <1:4, seroresponse was defined as a titer of ≥1:16; for participants with baseline hSBA titers ≥1:4 and <LLOQ (1:16 for the strain expressing fHbp variant A22; 1:8 for all other strains), seroresponse was defined as a titer ≥4 times the LLOQ; and for participants with baseline hSBA titers ≥LLOQ, seroresponse was defined as a ≥4-fold rise in titer from baseline.

[b]Composite responses were evaluated for serogroup B only and were defined as seroprotective titers (titers ≥LLOQ) for all 4 serogroup B strains combined.

[c]Serogroup B strains are indicated by the vaccine-heterologous fHbp variants they express.

[d]Data are for the post–dose 2 evaluable immunogenicity populations (Month 0,36 group, n=86–98; Month 0,12 group, n=110–116).

[e]Data are for the post–dose 1 evaluable immunogenicity populations (Month 0,36 group, n=141–143; Month 0,12 group, n=136–140).

[f]Data are for the post–dose 2 evaluable immunogenicity populations (Month 0,36 group, n=83–94; Month 0,12 group, n=111–116).
