# Supplementary material for: Immunogenicity and Safety of Extended Dosing Intervals for Pfizer Pentavalent MenABCWY Meningococcal Vaccination in Healthy Adolescents: Results from a Randomized, Phase 2b Study
Source: Vaccines (Basel). 2026 Apr 15;14(4):352. doi: 10.3390/vaccines14040352 (PMC13120601; doi:10.3390/vaccines14040352)
Supplement: Supplementary file 1 [file vaccines-14-00352-s001.zip › vaccines-4041683_Table S6.pdf]

Table S6. hSBA GMTs Against Serogroup B Strains and Serogroups A, C, W, and Y

	Strain[a]	Month 0,36 Group, GMT (95% CI)	Month 0,12 Group, GMT (95% CI)
Serogroup B			
Baseline[b]	A22	9.2 (8.4, 10.1)	9.0 (8.3, 9.9)
	A56	4.1 (3.9, 4.2)	4.1 (4.0, 4.3)
	B24	4.1 (4.0, 4.2)	4.1 (3.9, 4.4)
	B44	4.0 (4.0, 4.1)	4.0 (4.0, 4.1)
1 mo after second dose[c]	A22	99.5 (86.3, 114.6)	107.1 (93.3, 123.0)
	A56	315.6 (264.8, 376.2)	213.7 (180.8, 252.5)
	B24	47.0 (40.5, 54.7)	27.3 (23.9, 31.2)
	B44	132.6 (110.2, 159.6)	57.5 (48.4, 68.3)
Serogroups A, C, W, Y			
Baseline[d]	A	4.6 (4.1, 5.1)	4.4 (4.1, 4.7)
	C	4.7 (4.2, 5.2)	4.4 (4.1, 4.6)
	W	4.8 (4.4, 5.3)	4.9 (4.5, 5.5)
	Y	6.8 (5.6, 8.1)	6.3 (5.5, 7.3)
1 mo after first dose[e]	A	147.9 (124.8, 175.2)	133.2 (111.1, 159.6)
	C	41.8 (30.8, 56.7)	51.5 (36.5, 72.6)
	W	47.7 (39.4, 57.7)	48.0 (39.2, 58.8)
	Y	80.8 (65.5, 99.6)	80.0 (65.8, 97.2)
1 mo after second dose[f]	A	439.3 (367.4, 525.2)	236.9 (203.7, 275.5)
	C	401.9 (328.6, 491.4)	140.0 (114.0, 172.0)
	W	1040.1 (871.0, 1242.0)	385.7 (327.5, 454.2)
	Y	876.3 (715.6, 1073.2)	377.8 (315.7, 452.1)

fHbp=factor H binding protein; GMT=geometric mean titer; hSBA=serum bactericidal assay using human complement.

Corresponding data are in **Figure 4**.

[a]Serogroup B strains are indicated by the vaccine-heterologous fHbp variants they express.

[b]Data are for the post–dose 2 evaluable immunogenicity populations (Month 0,36 group, n=90–99; Month 0,12 group, n=114–116; 0-,6-month schedule, n=831–849).

[c]Data are for the post–dose 2 evaluable immunogenicity populations (Month 0,36 group, n=96–99; Month 0,12 group, n=113–116; 0-,6-month schedule, n=794–847).

[d]Data are for the post–dose 2 evaluable immunogenicity populations (Month 0,36 group, n=98–100; Month 0,12 group, n=113–116; 0-,6-month schedule, n=439–451).

[e]Data are for the post–dose 1 evaluable immunogenicity populations (Month 0,36 group, n=143–144; Month 0,12 group, n=140; 0-,6-month schedule, n=507–509).

[g]Data are for the post–dose 2 evaluable immunogenicity populations (Month 0,36 group, n=83–95; Month 0,12 group, n=114–116; 0-,6-month schedule, n=455).
