# Supplementary material for: Immunogenicity and Safety of Extended Dosing Intervals for Pfizer Pentavalent MenABCWY Meningococcal Vaccination in Healthy Adolescents: Results from a Randomized, Phase 2b Study
Source: Vaccines (Basel). 2026 Apr 15;14(4):352. doi: 10.3390/vaccines14040352 (PMC13120601; doi:10.3390/vaccines14040352)
Supplement: Supplementary file 1 [file vaccines-14-00352-s001.zip › vaccines-4041683_Table S7.pdf]

Table S7. Details of SAEs of Psychiatric Conditions Occurring Within 6 Months of Study Vaccination

Vaccination Group	Participant demographics and characteristics	Baseline Medical History	First Pfizer MenABCWY Dose	Second Pfizer MenABCWY Dose	SAE	SAE date (time since last study vaccination)	Outcome	Causality[a]
Month 0,12	Age at enrollment: 11 years<br>Sex: Female<br>Race; Ethnicity: White; Non-Hispanic /Latino	None	Administered	Administered approximately 12 months after the first dose	Intentional overdose[b]	186 days after second MenABCWY dose	Resolved	Not related to Pfizer MenABCWY
Month 0,36	Age at enrollment: 11 years<br>Sex: Female<br>Race; Ethnicity: White; Non-Hispanic /Latino	None	Administered	Participant was withdrawn from the study due to suicidal ideation (as no longer met eligibility criteria) and did not receive a second MenABCWY dose	Suicidal ideation	472 days after only MenABCWY dose	Resolved	Not related to Pfizer MenABCWY

SAE, serious adverse event.

[a]As assessed by the study investigator.

[b]Following the intentional overdose, the participant subsequently experienced an acute hepatic failure SAE deemed unrelated to vaccine; the acute hepatic failure event later resolved.
