# Supplementary material for: Immunogenicity and Safety of Extended Dosing Intervals for Pfizer Pentavalent MenABCWY Meningococcal Vaccination in Healthy Adolescents: Results from a Randomized, Phase 2b Study
Source: Vaccines (Basel). 2026 Apr 15;14(4):352. doi: 10.3390/vaccines14040352 (PMC13120601; doi:10.3390/vaccines14040352)
Supplement: Supplementary file 1 [file vaccines-14-00352-s001.zip › vaccines-4041683_Table S8.pdf]

Table S8. hSBA GMTs and Percentages of Participants with Seroprotective hSBA Titers, hSBA titers ≥1:4, and hSBA Seroresponses Against Serogroup B and Serogroups A, C, W, and Y for the 0-,6-Month Pfizer MenABCWY Vaccine Schedule[a]

	**Strain**[b]	**GMTs, % (95% CI)**	**Seroprotective titers,**[c] **% (95% CI)**	**hSBA titers ≥1:4, % (95% CI)**	**Seroresponses,**[d] **% (95% CI)**
Serogroup B					
Baseline	A22	10.3 (9.9, 10.8)	17.0 (14.5, 19.8)	20.5 (17.8, 23.4)	-
	A56	5.1 (4.9, 5.4)	10.7 (8.7, 13.0)	13.6 (11.3, 16.1)	-
	B24	4.3 (4.2, 4.4)	5.2 (3.8, 6.9)	7.9 (6.2, 9.9)	-
	B44	4.2 (4.1, 4.3)	3.7 (2.5, 5.1)	6.4 (4.8, 8.2)	-
1 mo after second dose	A22	65.4 (61.1, 70.0)	92.2 (90.1, 94.0)	92.4 (90.4, 94.2)	83.0 (80.2, 85.6)
	A56	182.0 (169.5, 195.4)	98.7 (97.6, 99.3)	98.7 (97.6, 99.3)	95.9 (94.3, 97.2)
	B24	17.8 (16.7, 19.0)	83.4 (80.7, 85.8)	88.3 (85.9, 90.4)	68.1 (64.8, 71.2)
	B44	33.9 (31.6, 36.3)	94.3 (92.6, 95.8)	96.8 (95.4, 97.9)	86.5 (84.0, 88.7)
	Composite[e]	-	-	-	78.3 (75.2, 81.2)
Serogroups A, C, W, Y					
Baseline	A	5.6 (5.1, 6.0)	17.9 (14.5, 21.8)	-	-
	C	6.2 (5.7, 6.7)	30.6 (26.4, 35.1)	-	-
	W	7.6 (7.0, 8.3)	41.0 (36.4, 45.8)	-	-
	Y	10.0 (8.9, 11.1)	48.0 (43.3, 52.7)	-	-
1 mo after first dose	A	138.5 (125.1, 153.4)	99.4 (98.3, 99.9)	-	97.0 (95.1, 98.3)
	C	40.2 (34.2, 47.2)	82.4 (78.8, 85.7)	-	62.9 (58.5, 67.1)
	W	61.2 (54.8, 68.4)	98.8 (97.5, 99.6)	-	79.3 (75.4, 82.8)
	Y	99.8 (90.2, 110.4)	99.2 (98.0, 99.8)	-	82.0 (78.3, 85.3)
1 mo after second dose	A	171.0 (156.0, 187.4)	99.8 (98.8, 100)	-	97.8 (95.9, 98.9)
	C	145.9 (131.2, 162.3)	99.1 (97.8, 99.8)	-	93.3 (90.6, 95.5)
	W	263.1 (240.1, 288.3)	99.8 (98.8, 100)	-	97.3 (95.3, 98.6)
	Y	276.7 (253.6, 301.9)	99.6 (98.4, 99.9)	-	94.4 (91.8, 96.3)

fHbp=factor H binding protein; hSBA=serum bactericidal assay using human complement; LLOQ=lower limit of quantitation.

[a]Results are from a separate study that included individuals 10–25 years of age; only data for participants naive to the meningococcal vaccine(s) for the corresponding serogroup are presented [18].

[b]Serogroup B strains are indicated by the vaccine-heterologous fHbp variants they express.
[c]Seroprotective titers defined as hSBA titers ≥LLOQ (1:16 for the strain expressing fHbp variant A22; 1:8 for all other strains).
[d]For participants with baseline hSBA titers <1:4, seroresponse was defined as a titer of ≥1:16; for participants with baseline hSBA titers ≥1:4 and <LLOQ (1:16 for the strain expressing fHbp variant A22; 1:8 for all other strains), seroresponse was defined as a titer ≥4 times the LLOQ; and for participants with baseline hSBA titers ≥LLOQ, seroresponse was defined as a ≥4-fold rise in titer from baseline.
[e]Composite responses were evaluated for serogroup B only and were defined as seroprotective titers (titers ≥LLOQ) for all 4 serogroup B strains combined.
