# Supplementary material for: Immunogenicity and Safety of Extended Dosing Intervals for Pfizer Pentavalent MenABCWY Meningococcal Vaccination in Healthy Adolescents: Results from a Randomized, Phase 2b Study
Source: Vaccines (Basel). 2026 Apr 15;14(4):352. doi: 10.3390/vaccines14040352 (PMC13120601; doi:10.3390/vaccines14040352)
Supplement: Supplementary file 1 [file vaccines-14-00352-s001.zip › vaccines-4041683_Table S9.pdf]

Table S9. Percentages of Participants with Seroprotective hSBA Titers[a] Against Serogroup B and Serogroup A, C, W, and Y Strains Through 24 Months After the Second Vaccine Dose for the 0-,6-Month Pfizer MenABCWY Vaccine Schedule[b]

**Strain**	**Baseline, % (95% CI)**	**1 month, % (95% CI)**	**12 months, % (95% CI)**	**24 months, % (95% CI)**
Serogroup B[c]				
A22	28.6 (22.6, 35.3)	91.5 (86.9, 94.9)	32.7 (25.6, 40.5)	36.7 (30.0, 43.9)
A56	13.6 (9.2, 19.0)	97.6 (94.5, 99.2)	33.3 (26.1, 41.2)	34.7 (28.1, 41.8)
B24	11.0 (7.1, 16.0)	85.9 (80.3, 90.3)	30.9 (24.0, 38.6)	33.2 (26.6, 40.2)
B44	2.9 (1.1, 6.1)	95.3 (91.5, 97.7)	18.7 (13.1, 25.4)	18.0 (12.9, 24.0)
Serogroups A, C, W, Y				
A	10.8 (5.7, 18.1)	100 (96.8, 100)	91.1 (84.2, 95.6)	88.1 (80.2, 93.7)
C	29.4 (21.0, 38.8)	100.0 (96.7, 100)	76.8 (67.9, 84.2)	75.2 (65.7, 83.3)
W	25.7 (17.8, 34.9)	100.0 (96.8, 100)	99.1 (95.1, 100)	99.0 (94.7, 100)
Y	54.1 (44.3, 63.7)	100.0 (96.8, 100)	100.0 (96.8, 100)	100.0 (96.4, 100)

fHbp=factor H binding protein; hSBA=serum bactericidal assay using human complement; LLOQ=lower limit of quantitation.

[a]Seroprotective titers were defined as hSBA titers ≥LLOQ (1:16 for the strain expressing fHbp variant A22; 1:8 for all other strains).

[b]Results for are from a separate study that included individuals 10–25 years of age; only data for participants naive to the meningococcal vaccine(s) for the corresponding serogroup are presented [18].

[c]Serogroup B strains are indicated by the vaccine-heterologous fHbp variants they express.
