# Supplementary figures and images for: Immunogenicity and Safety of Extended Dosing Intervals for Pfizer Pentavalent MenABCWY Meningococcal Vaccination in Healthy Adolescents: Results from a Randomized, Phase 2b Study
Source: Vaccines (Basel). 2026 Apr 15;14(4):352. doi: 10.3390/vaccines14040352 (PMC13120601; doi:10.3390/vaccines14040352)

Supplementary Appendix

Figure S1. Study Design Overview

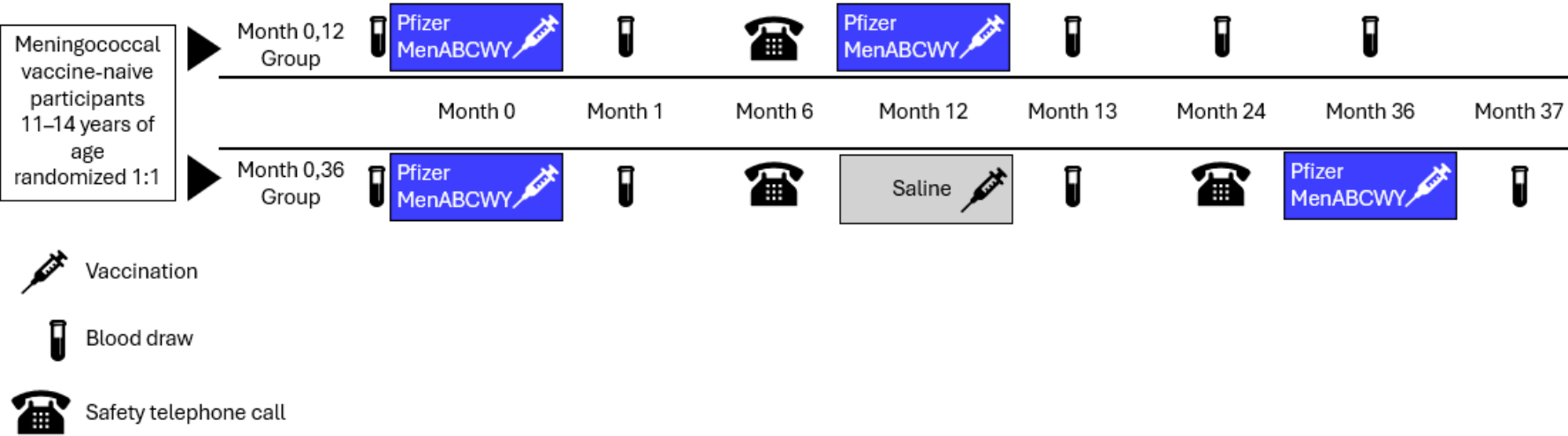

Supplement: Supplementary file 1 [file vaccines-14-00352-s001.zip › vaccines-4041683_Figure S1.pdf]
